# Short and Long-Term Effects of Growth Hormone in Children and Adolescents With GH Deficiency

**DOI:** 10.3389/fendo.2021.720419

**Published:** 2021-09-01

**Authors:** Michael B. Ranke

**Affiliations:** Children’s Hospital, University of Tuebingen, Tuebingen, Germany

**Keywords:** growth hormone deficiency (GHD), diagnosis, childhood, puberty, GH treatment, adult height

## Abstract

The syndrome of impaired GH secretion (GH deficiency) in childhood and adolescence had been identified at the end of the 19^th^ century. Its non-acquired variant (naGHD) is, at childhood onset, a rare syndrome of multiple etiologies, predominantly characterized by severe and permanent growth failure culminating in short stature. It is still difficult to diagnose GHD and, in particular, to ascertain impaired GH secretion in comparison to levels in normally-growing children. The debate on what constitutes an optimal diagnostic process continues. Treatment of the GH deficit *via* replacement with cadaveric pituitary human GH (pit-hGH) had first been demonstrated in 1958, and opened an era of therapeutic possibilities, albeit for a limited number of patients. In 1985, the era of recombinant hGH (r-hGH) began: unlimited supply meant that substantial long-term experience could be gained, with greater focus on efficacy, safety and costs. However, even today, the results of current treatment regimes indicate that there is still a substantial fraction of children who do not achieve adult height within the normal range. Renewed evaluation of height outcomes in childhood-onset naGHD is required for a better understanding of the underlying causes, whereby the role of various factors - diagnostics, treatment modalities, mode of treatment evaluation - during the important phases of child growth - infancy, childhood and puberty - are further explored.

## Introduction

The fundamental findings relating to the chemical structure of pituitary growth hormone and its biological effects on growth and metabolism in various animals were described in the first half of the 20^th^ century (1). The major driving forces in this field were Herbert Evans and his collaborators (2). By the beginning of the next half of the century, when the species specificity of primate GH in humans had been discovered and methods to purify GH from pituitaries of men and monkeys had been refined, the first studies to prove the efficacy of this peptide hormone were conducted. In 1958, human pituitary GH (pit-hGH) was shown to promote growth in a GH-deficient adolescent over a period of several months (3). Human and monkey pituitary GH revealed a variety of short term (days) metabolic effects in adolescents and adults with hypopituitary disorders (4). The era of pit-hGH ended in 1985, when hGH produced *via* recombinant technology became available. This initiated the era of virtually unlimited availability of r-hGH worldwide and the expansion of its use in adults with GHD, in children with growth disorders and for other indications.

The primary aim of this article is to review the effect of GH treatment on growth, predominantly in children and adolescents with GHD and to evaluate our current understanding of the factors affecting the magnitude of the response in the short- and long-term. Such an evaluation not only requires a review of the specific literature pertaining to treated cohorts but also necessitates a discussion – from a historical perspective – of the instruments and their suitability in establishing the diagnosis of GHD, along with the tools used to analyze the growth response during different developmental phases.

## Classification of Growth Hormone Deficiency

By definition, GHD is a syndrome caused by the impaired secretion of GH. This can be the consequence of a disorder at the level of the pituitary itself and/or within the cascade of function and structures of the hypothalamus or brain which regulate its secretion. However, the wider understanding of the term GHD also includes disorders resulting from impaired action of GH at the cellular level. After recognizing that GH-dependent components of the IGF-family were involved in mediating the effects of GH, the concept was nurtured that IGF was at the center of a GH–IGF regulatory system (5). On the basis on this concept, a distinction between *secondary* IGF-deficiency [IGFD] (as in GHD) and *primary* IGFD (=non-GHD) was proposed (6, 7).

Although this was a logical approach and suited for the clinical sub-classification of the GHD syndrome, it was simplistic and did not do justice to the complexity of the IGF-system (5–14). The major peptides of the IGF system in blood - IGF-I, IGFBP-3 and ALS - are GH-dependent and their levels in blood are quantitatively related to the GH secreted (15). But their levels in blood are also dependent on many other factors, for instance, hormonal or nutritional status (16, 17). In addition, growth promotion at the cellular level of the epiphyseal growth plate requires the local presence of both IGF and GH, whose quantitative relationship with their circulating levels is not fully understood (11, 12).

From the clinical perspective, it needs to be understood that GHD is also classified according to descriptive characteristics rather than a uniform principle (7). Some examples are:

•the onset of its origin: congenital/non-acquired *vs*. acquired;
•the hormonal extent of a pituitary defect: isolated (GH deficiency only) *vs*. combined [with other pituitary hormone deficits];
•the known cause: causal [specific cause known] *vs*. idiopathic [cause unknown];
•the extent of GH impairment: complete *vs*. incomplete (partial);
•its existence over the lifespan: permanent *vs*. transient;
•the age of disease discovery: during infancy, childhood, adolescence, or adult life.

## Prevalence – Incidence of GHD

Reports on the incidence or prevalence of GHD in children are scarce. In the pit-hGH era, when very short children (height < -3 SDS) used to be diagnosed and a GH cut-off to tests of < 5 ng/mL was applied, an incidence of 1:4000 and a prevalence of 1:5,000-30,000 were reported (18–20). During the r-hGH era, when the test cut-off was at 10 ng/ml, an incidence of 1:3,400 and a prevalence of 1:29,000 were reported (21). After r-hGH became available in 1987, a doubling of the incidence in childhood-onset GHD in Denmark was observed, which was similar to that in southern Germany (22, 23).

## Diagnosing GHD in Children and Adolescents

There is no single tool to confirm GHD. Thus, the diagnosis must be established by means of a variety of symptoms, signs and test results. The interpretation of non-clinical investigations must always be in accordance with clinical findings. Quantitative results need to be based on methodologically correct procedures and must be compared with appropriate normative references. Abnormal test results should always be repeated, particularly if they do not correspond with other findings.

The diagnostic path to establishing GHD involves several steps:

•history (family, gestation and birth, individual),
•clinical investigation,
•anthropometrical (growth) evaluation,
•static biochemical tests,
•GH-related basic biochemical investigations,
•evaluation of GH secretion,
•imaging techniques,
•molecular genetics.

Commonly, the initial suspicion of GHD is proposed by a general practitioner or family physician, who observes signs of impaired growth; while the conclusive diagnosis of GHD is confirmed by paediatric endocrinologists in tertiary institutions. Therefore, in some medical environments, the criteria for referring such children from a lower level of child care to experts may differ from the criteria used by specialists to confirm GHD (24, 25).

Due to the complexity related to diagnosing and classifying GHD in childhood and adolescence, in particular in the less severe, non-acquired forms, a number of controversies had arisen which led to numerous publications by specialists, societies and expert groups over the years (26–30). In the author’s view, there are only a few aspects which are of particular significance in diagnosing and treating GHD during the main phases of growth - infancy, childhood, and puberty (which partly overlap) (31) – these will be considered in detail here.

## GHD in Infancy and Very Early Childhood

While – in simplistic terms - postnatal growth during the childhood phase is apparently driven by parameters of the GH-IGF system, prenatal growth is primarily influenced by the insulin-nutrition environment. During the first months of life, GH blood levels are high, while those of IGF-I are low, presumably due to lower GH sensitivity during the growth phase of infancy, which, when it fades, is accompanied by an inverse trend: decline of GH and increase in levels of GH-dependent hormones (e.g., IGF-I, IGFBP-3) (32, 33). The dynamics of growth and the GH-IGF system during infancy and early childhood pose specific problems when diagnosing GHD during this period of life. In contrast to later childhood, the suspicion of GHD in the neonatal period is commonly neither driven by severe smallness at birth (34) nor by poor postnatal growth, but often by normo-insulinemic hypoglycaemia or/and protracted postnatal icterus (with elevated direct bilirubin), or/and underdeveloped external genitalia (phallus, clitoris, maldescensus testis). Besides hypoglycaemia, the other signs are commonly only present in the additional absence (also prenatally) of other pituitary (TSH, LH, FSH, ACTH) hormones.

Although conventional techniques to quantify GHD secretion as described below are generally not applicable during this phase of life, the diagnosis of GHD in suspected cases can be established without dynamic tests. Indications of GHD can be ascertained by means of basal IGF-I measurements and/or IGFBP-3 of < - 2 SD (sensitivity of 80%) (35) and *via* tests of GH levels using single serum drawn during hypoglycaemia (GH < 20 ng/mL) (36). In infants and toddlers very low normal levels of IGF-I make it difficult to distinguish normal from GHD (16). Therefore IGFBP-3 is the preferred diagnostic tool at this age. Additionally, filter paper samples used for neonatal screening also offer clues (GH < 7 ng/mL) (37), as does a series of low, randomly-measured GH levels.

Growth in infancy is very dynamic: body length at 2 years is about 40 cm greater than at birth. Height velocity (HV) decreases from about 25 cm/year during the first year to about 12 cm/year during the second year (38). About 50% of infants with congenital GHD deviate from the infancy component of growth (39) and height after one year declines below normal limits (40). However in many cases in which GHD was detected during childhood, low height velocity could have previously been observed in infancy (41, 42). On the other hand, feeding difficulties and failure to thrive may be misleading symptoms in terms of GHD. The careful evaluation of length and weight during regular post-natal care could thus lead to an increase in the fraction of children with suspected/diagnosed GHD at an early age.

Children who are diagnosed very early in life often suffer from a congenital disorder (cGHD), such as anatomical defects in the hypothalamic-pituitary region (e.g., pituitary stalk interruption syndrome [PSIS]), which can be visualized by means of neuroimaging (43) or by identifying other genetically-caused disorders (44, 45). Such cases are often associated with combined pituitary hormone deficiencies. Whether or not perinatal head trauma is a possibly relevant cause of GHD acquired at birth, as suggested in the past (46), is yet to be clarified. Differences in the characteristics of very young children with GHD as compared to those during childhood have been documented in a few series (47–49) and are listed in **Table 1**.

**Table 1:** Characteristics of very early onset of GHD compared to childhood onset.

Age group		0-1 year	0-3 years	0-2 years	6-8 years
Authors		Huet et al. (1999) (47)	Cetinkaya et al. (2017) (48)	Ranke et al. (2003) (49)	
N (m/f)		59 (33/26)	67 (37/30)	234 (154/80)	1,498 (1.004/494)
Birth Length	SDS+	-0.9	-1.0	-0.6	-0.5
Breech delivery	%	–	6	10.7	4.8
Age	yrs*	0.5^+^	1.2^+^	1.4	6.9
Bone Age	yrs*	–	–	0.8	4.5
Length/Height (Ht)	SDSCA+	-3.5^+^ +/-1.9	-3.9 +/-1.3	-3.5	-2.4
Ht - tHt	SDSCA+	-3.1	–	-3.3	-1.8
Test: maxGH	ng/mL*	2.2^+^	1.0 (0-6.5)	4.0	6.5
Hypoglycemia	%	85	–	30	3
Microphallus	%	52^&^	–	28	2
Isolated GHD	%	15	25	50	86

*median; ^+^mean; ^&^male only.

## Non-Acquired GHD During Childhood

### Anthropometry

It is the observed deviation from normal growth – from about two years of age to the onset of puberty – that typically initiates exploratory steps towards diagnosing GHD. A comprehensive analysis of growth must include measurements of height, weight, head circumference, and other anthropometrical data to determine body proportions (e.g., sitting height, arm span); in addition, it is also imperative to apply methods to estimate the relative amount of fat mass (e.g., BMI, fat fold thickness, DXA). In order to visualize and/or calculate the extent of any deviation from normal values, appropriate references need to be applied. For the assessment of height, there are up-to-date and ethnically-appropriate references, which are commonly available for the corresponding population; and, in parallel, SD scores for chronological age (Ht SDS_CA_) should be calculated. By convention, a height measurement below –2.0 SDS_CA_ defines short stature for a given population. In order to determine height in relationship to parental height, a familial “target height” must be calculated and transformed into an SD-score (THt SDS) based on the same references (50, 51). This information is then used to calculate the child’s height, corrected for its parental target height (cHt SDS_CA_ = Ht SDS_CA_ - THt SDS). A cHt below –1.3 SDS_CA_ (equivalent to about the 10th centile), roughly denotes shortness outside the familial range. It is remarkable many recent national guidelines do not recommend cHT as a diagnostic criterium (25).

Height velocity [HV] - the change of height over time (cm/year) – expresses the dynamic growth process and is considered the “golden parameter” for any growth evaluation. However the calculation of HV necessitates taking a minimum of two height measurements in 3, 6 and 12-month intervals. The time required between two measurements, in order to obtain an accurate result, is a function of the underlying HV [the greater, the shorter] and the error of Ht measurement [the smaller, the shorter]. The HV SDS_CA_ is calculated on the basis of appropriate numerical HV references, deriving from (difficult-to-obtain) longitudinal investigations (52). Moreover, the complex dynamics of height velocity over time, plus the common delay in developmental tempo in GHD, as evidenced by a delay in bone age [BA], makes HV - and even more so HV SDS_CA_ - a diagnostic tool prone to error. Therefore, it is difficult to clearly distinguish between normal HV and one that is too low in children with suspected GHD. However, a HV SDS_CA_ > -1.0 SDS (approx. 25th centile) is considered to be unlikely during childhood, in the context of non-acquired GHD (25, 26). A practical and probably more robust surrogate measure for HV is the change in height, expressed in terms of delta HtSDS_CA_, derived from two Ht measurements taken 6-12 months apart. A decrease in delta Ht SDS (deflection) of >0.25 SD over one year is considered to be a strong indicator of true growth disorder during childhood (53, 54). Since the diagnostic procedure for childhood non-acquired GHD often takes several months, and considering that height measurements were frequently documented in the past, it became evident that the inclusion of HV parameters strengthens the diagnostic process without unduly delaying treatment.

The appearance of a child with severe GHD can be conspicuous: there may be puppet-like features, with a relatively large neurocranium, slight truncal obesity, and small hands and feet, among other characteristics. However less attention has been given to the measurement of various relevant anthropometrical features and to compare them with the height data (in terms of SDS_CA_) of normal and short children (55, 56). Only few comprehensive references have documented a great variety of anthropometrical variables in children simultaneously (38, 57). Although such references may not match the population of the child in question, they need to be applied in order to ensure complex anthropometrical analyses. If different normative references for each parameter (e.g., height, weight, arm span) are used in calculating SD scores, a false picture will emerge. Investigations of body composition with the help of modern tools, such as DXA, BIA, CT and MRI, provide evidence of the negative change in the muscle to fat mass ratio that is typical for GHD children.

An x-ray of the hand and wrist is done to evaluate bone maturity [transformed into bone age (BA)]. If possible, it should be determined automatically in order to avoid a rater bias (58), but also to detect a primary bone disorder, as part of the evaluation for GHD. It is important to remember that, in GHD, a BA [yrs] > (CA – 1) [yrs] is not likely to be found in true GHD during childhood (59, 60).

### Insulin-Like Growth Factors

The two most important GH-dependent static peptide hormones in blood that must be measured during the diagnostic work-up of GHD are insulin-like growth factor-1 (IGF-I) and the IGF-binding protein-3 (IGFBP-3). They are part of a complex system that regulates cellular growth (13). The immunoassay is a well-established method for measuring these peptides in body fluids (61, 62) and reference values of basal blood levels over the whole human age spectrum in both sexes have been established by means of various assays (16, 63, 64). Based on the results of IGF levels in blood, further GH testing may be required in short children in order to obtain compelling evidence for the true existence of GHD. The interpretation of IGF levels measured by means of this biochemical diagnostic process must include the results of the above-mentioned clinical and anthropometrical investigations (65).

There is a wealth of literature on the diagnostic utility of IGF-I and/or IGFBP-3 measurements in the case of childhood GHD (16, 66, 67). In most of these studies, groups of children with GHD, based on various results of diagnostic tests, were analyzed. The IGF results in groups with (often isolated idiopathic) GHD were compared with groups of children with similar clinical characteristics but who had been classified as non-GHD (e.g., idiopathic short stature [ISS] (68, 69). The criteria for the anthropometric work-up and the static biochemistry in the studies with patients during mid- to late childhood were not uniform; in addition, the modalities of GH quantification (assays, test procedures) and cut-off levels to tests (commonly between 5 and 10 ng/mL) varied between studies. Nevertheless, the overall results from studies in which a cut-off of 10 ng/mL of GH (maximum) was implemented show a rather uniform qualitative picture: For both IGF-I and IGFBP-3 (expressed as an SD score for age), a cut-off of about -2.0 SDS denoted lower sensitivity (the power to correctly confirm GHD) than specificity (the power to correctly exclude GHD) (16). Thus a normal level is likely to exclude GHD, but below normal levels do not prove GHD. When a GH test cut-off of 7-8 ng/mL was accepted as evidence of GHD in childhood, IGF-I levels of < -1.4 SDS demonstrated a sensitivity of 100% and a specificity of 33%. In the same cohort investigated, a IGFBP-3 level of < -0.2 SDS showed a sensitivity of 100% at a specificity of 14% (70). In many countries, an IGF-I level of < - 2.0 SDS is a requirement for the diagnosis of GHD during childhood (25). However, a note of caution should be given here: the reference ranges reported for children ensued from a number of different assays, which is why the derived SDS_CA_ values of IGF-I or IGFBP-3 may differ considerably. New approaches for establishing multidimensional references may be developed in the future (17).

## Defining Impaired hGH Secretion

The core issue for the diagnosis of GHD is to obtain proof of impaired GH secretion. This entails determining hGH in blood as well as exactly quantifying GH secretion in normal and short children. The possibility to measure minute quantities of hGH in blood, for clinical purposes, started with the first immunoassays in 1963; and a process of methodological refinement has followed ever since (71–73). This process has involved, among others, the development of international reference preparations - from pit-hGH [IRP 66/217; specific activity approx. 2 I.U./mg] to authentic r-hGH of the 22 kD variety [IRP 98/574, specific activity 3 I.U./mg] (74), in addition, it has advanced from the use of polyclonal antibodies to very specific monoclonal antibodies for (22 kD hGH) detection. Modern assays do not determine all GH variants, which may have biological functions different from 22 kD hGH (75).

The discovery of the pulsatility of pituitary GH secretion led to the recognition that it is not possible for single measurements to represent the overall amount of GH secreted. Consequently, the total daily amount of GH secreted began to be quantitated by means of various procedures over the whole age range (76, 77). Groups which used spontaneous GH secretion for the evaluation of the GH secretory status in children mostly took a frequent sampling approach (e.g., every 20 or 30 minutes) over 8-12 hours of sleep; and considered a maximum GH level of > 7 ng/mL and/or an integrated level of > 3 ng/mL) to be the approximate borders of normality in prepubertal children (70, 78). However this approach was not held to be feasible by most physicians involved in diagnosing GHD proper in pediatric endocrine practices (26). Nevertheless, the quantitation of spontaneous GH secretion remains a prerequisite for diagnosing one variety of GHD, namely, neurosecretory dysfunction (79).

The discovery that hypoglycemia can provoke a GH release, the magnitude of which can be taken as a surrogate for the secretion capacity (80, 81) initiated the identification of many such stimuli (36) which found their way into our clinical routine. However the mechanism of GH stimulation through such agents differs from their “stimulatory power”, due to the fact that their effects may also vary, depending on their susceptibility to metabolic and other influences (36, 82–84). In the search for a parameter that reveals normal/too low GH secretion in patients, clinicians opted for a plain and simple answer: the maximum level observed during a test. This set off the ongoing debate about “cut-off” levels, which basically depend on the GH assay and test procedure used. The low repeatability of all types of stimulation tests was acknowledged and the medical community agreed upon accepting only the maximum level of two tests in differentiating between GHD and non-GHD. In “standard” tests, a maximum level of >5- 10 ng/mL was accepted as normal in prepubertal children; on the other hand, it was recognized that test procedures involving GH-releasing hormone (GHRH) provoked a release of pituitary GH, which is about 2-4 fold higher than that seen in “classic” tests (85).

Since the amount of GH secreted spontaneously or through stimulation depends on other factors, such as age, sex, pubertal stage, body composition and nutritional stage; and also varies individually from day to day, it remains a very difficult task to establish normal references. Moreover, each child may also have an inherent set point of GH secretion for maintaining physiology. Thus, in order to define GHD in children by means of a complex diagnostic process, it is expedient to apply a cut-off range for GH levels rather than use a single cut-off.

## GHD: Diagnosis at Early Pubertal Age

### Anthropometry

At the time when puberty can be expected in normal children (86, 87), short children do not exhibit signs of puberty. Thus during this period, it is particularly difficult to differentiate between true GHD and idiopathic short stature (of the variety with pubertal delay) or hypogonadism (88). The diagnostic problems are mainly related to (1) establishing the onset of puberty, (2) the evaluation of growth, and (3) the issue of how to determine an impairment in GH secretion.

Tanner introduced the globally-used standards for the clinical stages of puberty (89). The onset of puberty in girls can be determined by palpating breast tissue, not by inspecting the breast, since breast tissue growth is an effect of estrogen. In boys, the onset of puberty is assumed at a mean testis volume of ≥4 ml, the volume being predominantly an indicator of an increase in the testicular seminiferous structures and not testosterone production. Testis volume is commonly estimated by comparison with an orchidometer (90). These procedures are prone to inaccuracies, which are not eliminated by applying new methods like sonography. The analysis of a pubertal growth spurt by means of mathematical algorithms (91–93) has shown that the onset (“take-off) of puberty - which is driven by hormones - is an exact indicator and may occur 6-36 months before the clinical signs mentioned above are evident.

For the diagnosis and quantification of a growth disorder, it particularly relevant to adequately compare an individual’s height with normative height references. According to historical data devised by Marshall and Tanner (86, 87), the pubertal stage B2 in girls normally occurs between about 8 und 13 years of age, whereas the pubertal stage G2 in boys normally occurs between about 10 and 14 years of age. The normal take-off of the pubertal growth spurt occurs at about 8-11 years of age in girls and 10-12 years of age in boys (38). Thus, in clinically prepubertal children, a height deviation from normal at pubertal age – expressed in terms of HtSDS_CA_ - is falsely exaggerated, since the normal growth curve has left the childhood path and is dominated by the pubertal component of growth (93). A Belgian survey showed that 19% of 295 children diagnosed with IGHD were ≥ 11 years of age; similar results - 17% of 156 children - were found in a German study (21, 23). In these children, height should rather be compared with data based on childhood references that have been extrapolated (adjusted) into the pubertal age range (94, 95). It is not known whether bone age - instead of CA – would be suitable to correct the error of HtSDS calculations based on CA. This aspect is even more relevant in terms of height velocity, for which adjusted HV references are available (96). Height velocity shows a marked prepubertal nadir which is more pronounced the longer puberty is delayed (38, 97). This is why, in the author’s view, a low HV should be interpreted with great caution in children during the pubertal age. These anthropometrical considerations can be effective in correcting the calculated growth parameters for delayed puberty and may increase the likelihood of classifying short children correctly before biochemical testing is done. For the static GH-dependent parameters, IGF-I and IGFBP-3, which also increase during hormonal puberty take-off, similar considerations should apply; in addition, adjusted references should be published in order to avoid the falsely low calculations of SD scores for age. This may avert inappropriate treatment being given on the basis of incorrect (false positive) classification of isolated naGHD during the pubertal age.

### Impaired GH Secretion and Priming

The next and even more strongly debated issue is the question of how to interpret GH test results during the pubertal age. Puberty onset varies between populations, but as discussed above, starts at the earliest at about 8 years in girls and 10 years in boys and is accompanied by marked hormonal changes (98, 99). We know today that the amount of GH secreted is augmented during puberty, as a result of estrogens secreted in both sexes (100). While there seems to be no major change in GH secretion during mid-childhood, the total amount of spontaneously secreted GH during puberty is increased (78) as are the maximal levels of GH observed in varying test procedures (36, 101, 102). Logically, this means that higher cut-off levels should mark subnormal GH secretion in pubertal (GHD) children. In contrast, a (short) child who is still prepubertal during the pubertal age may secrete GH amounts considered to be too low – but only on grounds of non-existing puberty. The same reasoning applies for the static IGF parameters that are not adapted for delayed puberty.

To avoid such misclassification, it was proposed that GH testing in these children should be conducted after exposing them to sex steroids (called “sex-steroid priming”) to briefly induce sex steroid augmented GH secretion (103). Unfortunately, this procedure, involving short exposure to estrogen (in girls) or aromatizable androgens or estrogen (in males), is not standardized. Nevertheless, it has been shown that such priming leads to enhanced maximal GH levels in tests (36, 103, 104). However the endocrine community is still divided on this issue (25, 26, 105, 106). It is likely that the wish to diagnose non-acquired GHD at pubertal age will diminish when the anthropometric and other tools mentioned above are valued for facilitating the correct interpretation of data in the context of naGHD. Some examples of characteristics of children at the timepoint of diagnosis, recorded over the past 50 years, are listed in **Table 2** (107–110).

**Table 2:** Characteristics of children and adolescents with non-acquired GHD (idiopathic GHD [IGHD] plus congenital GHD [cGHD]) at diagnosis).

hGH available		pit-Hgh National Institution			pit-hGH commercial	r-hGH		
Qualifying hGH Test Maximum		< 5.0 ng/mL			<7 ng/mL	< 10 ng/mL		<7-8 ng/mL
Author		Soyka et al. (1970) (Boston) (107)	Prader et al. (1970) (Zürich) (108)	Aceto et al. (1972) (USA) (109)	Ranke et al. (2018) (Tübingen) (110)			
Period -Years		<1970	1960-70	<1972	1968- 87	1988-97	1998-07	2008-15
Parameter								
N		15	7	52	87	112	331	45
Age (10th-90th centile)	yrs*	8.7	8.0	11.2	8.2 (4.0-15.3)	5.6 (2.9-11.9)	6.7 (4.1-13.5)	5.1 (2.5-10.6)
BoneAge	yrs*	na	4.6	5.9	4.4	3.8	4.8	4.2
Height (Ht)	SDS_CA_+	-5.0	-4.7	-5.8	-4.3	-3.3	-2.9	-3.1
Ht-velocity	cm/yr*	2.8	2.5	3.4	4.7	4.9	5.1	5.3
deltaHt	SDS_CA_+	na	na	na	-0.14^a^	-0.23^b^	-0.04^c^	-0.23^c^
Test: maxGH	ng/mL*	<3.1	na	<10	4.1	5.8	5.1	4.2
IGF-I	SDS_CA_+	na	na	na	-2.9	-3.2	-2.6	-4.8
IGFBP-3	SDS*CA*+	na	na	na	na	-2.7	-1.0	-3.4
Isolated GHD	%	na	na	na	40	63	77	82

^*^Median; ^+^Mean; comm, commercial production; ^a^n = 32; ^b^n = 52; ^c^n = 214; ^d^n = 36; na, not available.

## Treatment of GHD With hGH

### Aims of GH Treatment

In GHD, replacement with hGH aims at the normalization of deviant aspects of growth, body composition and body function. In children and adolescents, the issue of hGH efficacy is primarily associated with growth: rapid catch-up growth, normal maintenance growth, appropriate timing and magnitude of pubertal growth, and the achievement of an adult height within the normal range. In addition, efficacy in children with GHD should also include the achievement of normal body composition and functioning, as well as the normalization of biochemical abnormalities associated with GHD during post-adolescence and throughout adult life.

### Dosing and Mode of Application of hGH

The first patient to receive pit-hGH through Maurice Raben was initially given 1 mg, injected twice a week (b.i.w.) i.m.; later, the dose was raised to 3 mg, three times per week (t.i.w.). Raben administered his pit-hGH powder after reconstituting it in solvent (3). In subsequent years, pit-hGH units were devised, based on the growth response as well as the results of bio-assays using hypophysectomized female rats (111). More refined methods of purification led to a product with a potency of about 2 IU/mg (112). A dose effect in GHD – 5 IU b.i.w. *vs*. 10 IU b.i.w. - was observed by Preece et al. (113) Frazier described a linear-log relationship to the induced height velocity that resulted from doses ranging between at least 30 mIU/kg and 100 mIU/kg body weight t.i.w (114). The potency of recombinant hGH preparations was validated against international reference preparation with modern assays: 2.6 IU/mg for meth-r-hGH and 3.0 IU/mg authentic r-hGH. The amount of GH secreted - as evaluated by deconvolution analysis - was estimated to be about 20 µg/kg per body weight/day before puberty and about twice as high thereafter (76). The current starting doses of r-hGH, approved by authorities for prepubertal children, vary between a range of about 25-43 µg/kg body weight per day (115, 116) but may exceed this margin during puberty.

Pit-hGH was often administered using the total content of one ampule (2-4 IU), 2-3 times i.m. per week. After studies showed that the same amount could result in higher growth rates – in the long and short term - by dividing it into daily injections (117–119), daily s.c. injections became standard practice. GH doses are calculated either according to body weight (amount/kg BW) or per body surface (amount/m^2^ BS), with the latter precluding overdosing in obese patients. Today, exact doses can be applied easily with the help of “pens”, which may also allow monitored self-application (120). The role of long-acting GH variants for the treatment of GHD will be evaluated in the future (121).

### Adherence

Adherence (compliance) is an essential prerequisite for any therapy to be effective. The risk of non-adherence in GHD is high, because GH must be injected daily (by proxy or by patients) over many years. Great differences were found – mostly in short-term growth - in studies on this subject, particularly in terms of the method of recording adherence, the characteristics of the cohorts investigated and the quantification of missed injections (122–125). Generally, the level of adherence appears to be high during the important but less dose-dependent first year of treatment (126), but it is lower thereafter, particularly in independent adolescents (127). Even one missed dose per week during the first treatment year in children results in a loss of height gain of 0.11 SD (122), a number which adds up to a substantial figure over time. Due to the great heterogeneity of causes (e.g., discrepancy to expectation, social circumstances, injection problems), strategies to prevent non-adherence must be individually adapted (121, 124, 127, 128).

## Evaluation of the Growth Response and Results to GH Therapy in GHD

There have been roughly four phases of GH treatment from the time treatment with pit-hGH was first reported in 1958: (a) the experimental phase with pit-hGH (1958-approx. 1962), (b) the era of greater availability of pit-hGH (1962-1985), (c) the early era of r-hGH (1985–2000), and (d) the “consolidated” era of r-hGH (> 2000). The total growth process during GH treatment of GHD, starting with prepubertal age, can be divided into: (a) the initial phase of the first 2-3) years, which mark the phase of catch-up growth, (b) the childhood growth phase and (c) the pubertal growth phase, that ends in (d) the period in which (near) adult height is reached.

## Prepubertal Growth Phase

### Response Evaluation

The response to GH treatment is mostly analyzed in annual intervals and can be expressed in terms of height velocity (HV; cm/yr), change in HV in comparison to a previous period, HV SDS_CA_ and the resulting change (delta HV SDS_CA_) (129) or in terms of delta HT SDS_CA_ calculated over a certain period of time with treatment (prepubertal years, total puberty, start of GH to NAH). Pure HV (cm/yr) is a robust term and also practical as it can be visualized in a growth chart; however, it provides little exact information when measurements exceed the normal range. The expression of HV in terms of SD scores or changes over time is problematic, particularly during infancy and the pubertal age. During the catch-up phase and over longer periods of time, growth can also be described by means of mathematical algorithms (130–134).

Several cut-off levels for distinguishing between a normal and poor response during the first treatment year have been proposed: a change of ≥ 3 cm/year in HV as compared to pretreatment values (135), HV SDS ≥ mean – 1 SDS (136), HV SDS (for sex and age in normal children) ≥ + 1 SDS (52), delta Ht SDS ≥ +0.3 SDS or +0.5 SDS (137, 138). However comparisons led to inconsistent results (139).

### Empirical Response Targets

Rather than using normal references for evaluating the response to GH, it was proposed that results should be compared with the response of other treated patients. Based on large numbers of treated prepubertal children, who were observed in pharmaco-epidemiological surveys (NCGS and KIGS), references for HV (cm/yr) or delta Ht SDS were published (136, 138). These “height velocity targets (HVT)” took into consideration the diagnosis, sex, and age in prepubertal children from 4-13 years of age, but examined only the mean GH dose of the cohort. Based on NCGS data (136), HV targets were devised in graphical terms for both male and female children with IGHD and OGHD (maximum GH in tests: <10 ng/mL) for the first treatment year. The mean GH dose given was 0.30 mg/kg per week. Based on KIGS data (138) references for HV and delta Ht SDS were presented as graphs as well as numerically for prepubertal children with both severe (maximum GH in tests: < 5 ng/mL) and less severe (maximum GH in tests: 5-10 ng/mL) GHD, during the 1^st^ and 2^nd^ treatment year. The mean GH dose given was 0.22 mg/kg per week. The HVs of the GHD cohorts in the NCGS study were very similar to the HVs of the “severe” GHD cohort in KIGS.

### Growth Prediction

Another approach to evaluate the response of a treated patient is to compare the response variable (e.g., HV (cm/yr), delta Ht SDS) during a certain growth phase with the most likely expected response (and its error, at the start of each treatment phase) based on prediction algorithms derived from large cohorts. The advantage of this approach, as against using HVTs, is that validated prediction models consider a multitude of characteristics of an individual, the most important being the individual GH dose applied. The problem is to keep the error of prediction as low as possible. This error tends to rise when an increasing number of predictors that are not standardized are included. Several approaches have been used to develop prediction models (140–145). The observed and the predicted growth response can be compared and the difference can be expressed in terms of an ‘index of responsiveness’ (IOR) = [(observed response –predicted response)/error of prediction], which is a surrogate for the potential of an individual to respond (responsiveness) to GH, as compared to matched patients. An IoR below –1.0 denotes a poor response. Prediction models for various growth phases and diagnoses have been developed (145, 146) and are also available in the form of a software medical device (147). Prediction models will be developed further with the emerging field of pharmacogenomics (148, 149). Their applicability will expand with the growing importance of new electronic (self-)learning tools in medicine and in terms of optimizing cost-effective treatment.

Apart from the growth response, IGF-I targets have also been proposed as a means to guide and optimize dosing (150–153). Advocates of this approach point out that it offers a more cost-effective use of GH. Overall, the evaluation of the response to treatment, regardless of the tools used - particularly but not exclusively during the first phase of treatment - is of great importance in order to ensure an optimal outcome of growth in a treatment strategy which includes the prevention of non-adherence and an efficacious use of GH.

## Pubertal Growth Phase

Clinically, pubertal growth is the phase between the first appearance of clinical pubertal markers – breast in girls, testis volume in boys – and the end of growth due to the closure of the epiphyses of the long bones (89). In practice, the near end of growth is commonly assumed if the HV is below 2 cm/year and bone age is above 14 years in girls and 16 years in boys (154). Since hormonal changes take effect before clinical markers are noticeable, the pubertal growth phase is actually longer (95, 155, 156). Pubertal growth is governed by the interaction of sex steroids (estrogens and androgens in both sexes) with the activated GH-IGF system (100, 157) and its combined effects on the skeletal growth targets (158, 159). Several specific issues exist with respect to GHD treatment during pubertal growth: the GH dosing, the timing and length of puberty (starting age *vs*. end of growth) and the choice of sex steroid in the case of gonadotropin deficiency.

Bourguignon (160) discovered that total pubertal growth (TPG) is inversely correlated to age at onset of puberty in normal children, but that this did not affect final adult height. This means that the partial contribution of pubertal growth to total growth is inversely correlated to the prepubertal fraction. Accordingly, in idiopathic GHD (non-acquired GHD), TPG was found to be *positively* correlated with HT at puberty onset and at age at the end of growth and *negatively correlated* with age at puberty onset and that GH dose only has a minor effect (161). Mauras showed that a doubling of the prepubertal GH dose during puberty, over four years, results in only about 4 cm of additional gain in TPG (162). Thus, the extra gain in height by means of r-hGH during puberty is much more expensive. Results of studies comparing males and females with spontaneous or induced puberty showed a smaller gain in the induced groups, since they were older at puberty onset (**Table 3**) (163–165). However the lower pubertal gain in females is probably the result of sub-optimal estrogen replacement, in terms of timing, dose and preparation (166). Considering the fact that TPG only accounts for about 20% of total postnatal growth, the aim should be to normalize height well before puberty onset. It is a common observation that the relative height attained in terms of SD scores for age at puberty onset can be maintained even with prepubertal GH doses. Delaying puberty onset and prolonging the whole pubertal phase - with drugs suppressing puberty, such as GnRH (167) and/or increasing GH doses at puberty onset (e.g. doubling the dose over pre-pubertal levels) - are approaches to be considered in individual cases with non-acquired GHD as a kind of “rescue attempt” to improve adult height. By doing this, however, the well-known phenomenon of the acromegaloid phenotype of puberty may also be overly augmented.

**Table 3:** Examples of height in children with non-acquired GHD: start GH, puberty onset (spontaneous *vs*. induced), near adult height (NAH).

		Ranke et al. (1997) [KIGS] (163)				Thomas et al. (2001) [Belgium] (164)				Maghnie et al. (2006) [Italy] (165)			
		male		female		male		female		male		female	
		Pub spon	Pub ind.	Pub spon	Pub ind.	Pub spon	Pub ind.	Pub spon	Pub ind.	Pub spon	Pub ind.	Pub spon	Pub ind.
N		66	51	64	14	25	7	24	5	26	31	31	18
***GH start***		median				mean				median			
*maxGH to tests*	ng/mL	<10				<10				<10			
Age	yr	10.5	9.9	9.9	6.8	12.4	14.4	10.6	11.5	8.0	6.5	7.7	10.5
Ht	SDS_CA_	-2.7	-2.8	-2.9	-2.7	-2.7	-2.9	-2.7	-2.9	-3.0	-3.0	-2.6	-3.6
targtHt	SDS	-0.4	-0.7	-0.1	-0.4	-0.8	-0.1	-0.8	-0.1	-0.4	-0.5	-0.6	-0.4
GH dose	IU/kg	0.57				0.5-0.7				0.60			
GH (inj./wk)		r-hGH (2-7)				r-hGH (7)				r-hGH (5-7)			
***Pub start***													
Age	yr	13.8	14.9	12.9	13.7	13.3	17.2	11.8	14.9	13.4	14.9	12.6	13.5
Ht	SDS_CA_	-1.6	-1.3	-1.4	-1.0	-1.9	-1.4	-1.9	-1.4	-1.5	-2.3	-1.8	-2.3
Pub Ht gain	cm	22.5	19.6	15.0	10.4	27.5	17.1	22.2	9.6	22.8	20.5	17.1	16.5
***At NAH***													
Age	yr	17.8	19.2	16.0	17.0	19.1	21.0	16.2	18.5	17.6	19.4	16.5	20.0
Ht	SDS_CA_	-1.3	-0.5	-1.2	-0.9	-0.8	-0.3	-0.8	-0.3	-0.9	-0.7	-0.4	-0.8
Ht - Ht GHstart	SDS_CA_	1.3	2.3	1.7	1.7	1.9	2.6	1.9	2.6	2.1	2.3	2.3	1.7
Ht - Ht pub ons.	SDS_CA_	0.3	0.7	0.1	-0.1	0.0	0.1	0.0	0.1	0.6	0.6	1.4	1.5

Ht, height; Pub, puberty; spont., spontaneous; ind., induced; ons., onset.

## Adult Height Reached

Several reports were published after years of treatment with pit-hGH in which the adult height outcomes achieved in non-acquired GHD (often called IGHD) were described. These results were summarized in reviews (168–170). As exemplified in **Table 4**, (169, 171–174, 176) these patients had been severely GH deficient (maximum in tests < 7.5 ng/mL) and were relatively old (approx. mean age: 13 yrs) at diagnosis and GH start. These characteristics were not only due to a selection bias, since the oldest patients at start are the earliest to reach their (near) end of growth. On the other hand, the patients treated during the pit-hGH era were very short (mean height at GH start < -4.0 SDS) and were given dosages of about 8–12 IU of pit-hGH from various sources, injected 2-3 times per week i.m., and the total amount of one ampule often contained 4 (2) I.U. After about > 5-6 years of treatment, an adult height of about -3.0 SDS was reached in patients with spontaneous puberty, while those with induced puberty reached a height of about -1.5 SDS. Females tended to be younger and shorter at start but reached a lower adult height.

**Table 4:** Examples of groups of non-acquired GHD patients treated to NAH.

Authors		Wit et al. 1996 [review] (169)				Reiter et al. (2006) [KIGS] (176)				August et al. (1998) [NCGS] (171)		Rachmiel et al. (2007) [Canada] (172)		Westphal et al. (2008) [Sweden] (173)		Root et al. (2011) [GHD infant] (174)	
sex		m	f	m	f	m	f	m	f	m	f	m	f	m	f	m	f
GHD		Pub spon		Pub ind		iGHD		MPHD*		all		all		all		all	
N		131	31	97	30	351	200	257	172	153	195	73	23	294	107	23	24
*GH start*		mean				median				mean		mean		mean		mean	
maxGH	ng/mL	<7.5				<10				<10		<8.0		<10		<<10	
Age	yrs	12.8	11.6	13.8	13.5	10.1	9.3	8.0	7.2	12.0	10.9	12.4	10.4	9.1	8.0	0.8	1.0
Ht	SDSCA	-4.1	-5.1	-4.6	-4.3	-2.4	2.6	-2.9	-3.4	-2.6	-3.0	-2.8	-3.2	-2.7	-2.9	-2.4	-2.2
targetHt	SDS					-0.6	0.6	-0.3	-0.1	-0.5	-0.5	-0.4	-0.6	-1.2	-1.0	–	–
GH dose	IU/kg wk	0.2-0.5				0.6				0.9		0.54		0.7		0.9	
GH given		pit-hGH				r-hGH				met-r-hGH		r-hGH		r-hGH		met-r-hGH	
*At NAH*																	
Age	yrs	n.a.	n.a.	n.a.	n.a.	18.2	16.6	19.0	17.6	17.5	15.8	17.8	15.6	18.6	17.4	18.4	16.4
Ht	SDSCA	-3.1	-3.2	-1.5	-1.5	-0.8	-1.0	-0.7	-1.1	-1.3	-1.6	-1.0	-1.0	-0.9	-0.8	0.1	-0.8
Ht gain	SDS	1.3	1.9	3.0	2.7	1.6	1.6	2.3	2.3	1.3	1.4	1.7	2.1	1.8	2.1	2.3	1.4

Ht, height; Pub, Puberty; spon, spontaneous; ind, induced; GH dose, estimated from reports; iGHD, isolated GHD; MPHD*, multiple hormone deficiencies [induced puberty]; all, pituitary deficiencies combined; n.a., not available.

Patients during the early r-hGH era were reported to be less short (height about –2.9 SDS). With r-hGH doses of about 0.5 IU/mg/wk, injected in 3-7 fractions s.c. per week, they reached a height of about -1.4 SDS (164, 171, 175). As illustrated in **Table 4**, more recent patients who received somewhat higher doses and daily r-hGH injections reached a near adult height (NAH) mostly within the lower half of the normal range and closer to their calculated target height. However it should be remembered that in some populations there is a positive secular trend in adult height between generations in the order of about 0.4 SDS (54). Again, in such studies, females had a slightly lower height outcome. Japanese children with IGHD, who were treated with slightly lower doses compared to Europe/USA, achieved a slightly lower total height gain (176–178). Remarkably, practically all children treated as toddlers, for predominantly congenital organic GHD and MPHD, reached completely normal height (174).

Several authors have examined the factors correlating with NAH by means or regression analyses (116, 164, 165, 172, 173, 175). On the whole, the results of these studies revealed certain factors that correlated positively with NAH: height at GH start, mid-parental height, duration of treatment, GH dose, and the magnitude of the first year response to GH. On the other hand, the factors correlating negatively with NAH are: age at GH start and the severity of GHD (maxGH in tests, MPHD). In randomized studies, the long-term effect of GH dosage, in terms of NAH, was only marginally positive (179, 180). This may be due to the fact that childhood and pubertal growth are evaluated together: however, during childhood growth there is high sensitivity to GH, whereas during puberty there is low sensitivity to GH. The negative correlation of the outcome with the maximum GH level to testing may also suggest that the high GH cut-off may lead to the inclusion of non-/less severe GHD patients (e.g. ISS) who exhibit overall lower responsiveness to GH treatment. The negative effect of patients with MPHD is probably the result of an inappropriate induction and/or maintenance of puberty in children with gonadotropin deficiency. This needs further evaluation.

## Safety of hGH Replacement in Children

Safety issues during GH replacement may be related to the medical substance itself, may be due to the formulation of the drug (e.g. impurities, additives for drug formulation), be the result of the genuine (normal) effects (e.g., on the growth of bones, on other tissues, or be related to its metabolic action); they may be due to inappropriate dosages or a genuine incompatibility with the patient being treated (181). During the pit-hGH era, when relatively crude GH material were applied in low doses, local effects (pain, lipoatrophy) were occasionally observed (182). Due to the transmission of prions through some pit-hGH preparations, which caused the deadly Creutzfelt-Jakob disease, this era ended (183–185).

After the approval of r-hGH preparations, the analysis, detection and prevention of adverse effects became an integral part of large surveillance studies in children (186). Detailed reviews of the safety of r-hGH in children and adolescents are available (181, 187). A rare side-effect of normal GH action on accelerated bone growth in children is the slipped capital femoral epiphysis [SCFE] (188). The normal metabolic effects of sodium and water retention may cause benign intracranial hypertension (189). The anti-insulin effect of GH may cause impaired glucose tolerance or accelerate the development of DM2 in predisposed children with GHD (190). An early report associating pit-hGD with an increased risk of colonic cancer in GHD (191) raised a critical discussion about the potential role of the GH-IGF axis in cancer pathogenesis (192). A particularly controversial multinational survey on the safety and appropriateness of GH in Europe (SAGhE), which investigated mortality in adults who had received GH treatment in childhood, however presented inconclusive results (191, 193, 194). There is strong evidence that replacement with r-hGH in children and adolescents with non-acquired GHD is safe, as they receive the usual dosage range and have a low risk of other diseases (195, 196); nevertheless, it is prudent to ensure structured long-term follow-up and monitoring of IGF parameters during GH replacement (153, 187).

## Summary

For more than a century, it has been known that the growth hormone deficiency syndrome (GHD) affects the entire life span. Developments over many decades have led to the understanding of the key modalities, such as anthropometrical and biochemical methods, that facilitate the correct diagnosis of non-acquired – in particular isolated – GHD. However there are still a number of difficulties to overcome in order to arrive at the diagnosis as early and as properly as possible, particularly during the late childhood phase. The precise application of known techniques and principles in anthropometry as well as the prudent interpretation of test results is the imperative task of those entrusted with the medical care of children. During the past decades, replacement with GH has led to improvements in height gain during childhood and in final adult height. Yet a sizeable fraction of children does not achieve optimal adult height. Therefore the modalities for evaluating growth and the tools for adjusting treatment appropriately need to be further individualized and optimized, not only with regard to stature but also in terms of safety and costs. This entails combining the known principles of individual endocrine care with novel evidenced-based tools that substantiate the results of analyses before, during and after treatment.

## Author Contributions

The author confirms being the sole contributor of this work and has approved it for publication.

## Conflict of Interest

The author declares that the research was conducted in the absence of any commercial or financial relationships that could be construed as a potential conflict of interest.

## Publisher’s Note

All claims expressed in this article are solely those of the authors and do not necessarily represent those of their affiliated organizations, or those of the publisher, the editors and the reviewers. Any product that may be evaluated in this article, or claim that may be made by its manufacturer, is not guaranteed or endorsed by the publisher.
